# Supplementary material for: Chromothripsis during telomere crisis is independent of NHEJ, and consistent with a replicative origin
Source: Genome Res. 2019 May;29(5):737–49. doi: 10.1101/gr.240705.118 (PMC6499312; doi:10.1101/gr.240705.118)
Supplement: Supplemental Material [file supp_gr.240705.118_Supplemental_file_1.zip › contigs/annotated_contigs/DB48/contig.3.DB48_length_853_mean_cov_28.1043376319.docx]

**DB48_length_853_mean_cov_28.1043376319**

```
AGGAACAAACTCTTGGTCAAAAAGATTAATATCATAGGATCTCTAGCCAAATTCACCCTTAATAAATAAGTCCAGAGAGTAATTAAAAA
 >chr7:51689964-51690341 + E=7e-206 p=2e-02
AAAAAAACAAGAAAACATGGGGACCAGGTAGGATTAGGATGAAGGGCAGAGGGTTCTAGAACTATTTTTCTTTTGAAGCCTCTTCCATT

AGTGAGGATGTCTCCTTCATATTTATTTTTGGTGCTAGGTTTCATAGTGCTCAACACATAGACCTGGGCTGGAGGCTGTGGAGAAAGGG

CAGAGACCACATGCAGCTGTTTATCCCAAAAGTCAGTTTCCAAGAGCCGGCCTAACCCTGAAGGTGGATGGTTCAAAATATCTCATTTT

TATGAGAAATAAAGGTTC|A|AGACAAATAGATGGGAATTAATTAAACCAAAAAGCTTATGCCAGCAAAAGAAATAATCAGCAGAGTAA
                   >chr7:51606603-51606682 - E=1e-35
ACAGACA|ATT|TGCAGCAACCTAGGTGAAACTGGAGACCATTATTCCAAGTGAAGTAACTCAGGAATGGAAAACCAAACATTGTATGT
        >chr7:51605557-51605960 - E=1e-230
TCTTACTCATAAGTGGGAGCTAAGCTGTGAGGATACAGAGGAATAAGAATTTTACAATGGACTTTGGGGACACAGGGGGAAGGGTGGGA

GGGAGGTGAGGGATAAAAGACTGTACATTGGGAGGAATGTACACCGCTCAGGTGACTAGTGCACCAAAATCTCAGAAATCACCACTAAA

GAACTTATTCATGTAACCAAACACCACCTGTTCCCCAAAAACCTATTGAAATAAATAAAAGTAATAAAAATAAAAATAATTTTAAAAAA

CAAATACAGTCATACATCACTTAACAACTAAGATGTGCTCTGAGAAATGCATCATT
```
